# Supplementary material for: First Insights on Resistance and Virulence Potential of Escherichia coli from Captive Birds of Prey in Portugal
Source: Antibiotics (Basel). 2024 Apr 23;13(5):379. doi: 10.3390/antibiotics13050379 (PMC11117282; doi:10.3390/antibiotics13050379)
Supplement: Supplementary file 1 [file antibiotics-13-00379-s001.zip › antibiotics-2972817-supplementary.pdf]

Supplementary Material

First Insights on Resistance and Virulence Potential of *Escherichia coli* from Captive Birds of Prey in Portugal

Rita Magalhães [1,2], Raquel Abreu [1,2], Gonçalo Pereira [1,2], Eva Cunha [1,2,*], Elisabete Silva [1,2], Luís Tavares [1,2], Lélia Chambel [3] and Manuela Oliveira [1,2,4]

[1] CIISA—Centre for Interdisciplinary Research in Animal Health, Faculty of Veterinary Medicine, University of Lisbon, 1300-477 Lisbon, Portugal; rita-magalhaes@edu.ulisboa.pt (R.M.); rmsilva@fmv.ulisboa.pt (R.A.); goncalopereira@fmv.ulisboa.pt (G.P.); elisabetesilva@fmv.ulisboa.pt (E.S.); ltavares@fmv.ulisboa.pt (L.T.); moliveira@fmv.ulisboa.pt (M.O.)
[2] AL4AnimalS—Associate Laboratory for Animal and Veterinary Sciences, 1300-477 Lisbon, Portugal
[3] BioISI—Biosystems and Integrative Sciences Institute, Faculty of Sciences, University of Lisbon, 1749-016 Lisbon, Portugal; lmchambel@ciencias.ulisboa.pt
[4] cE3c—Centre for Ecology, Evolution and Environmental Changes and CHANGE—Global Change and Sustainability Institute, Faculty of Sciences, University of Lisbon, 1749-016 Lisbon, Portugal
* Correspondence: evacunha@fmv.ulisboa.pt

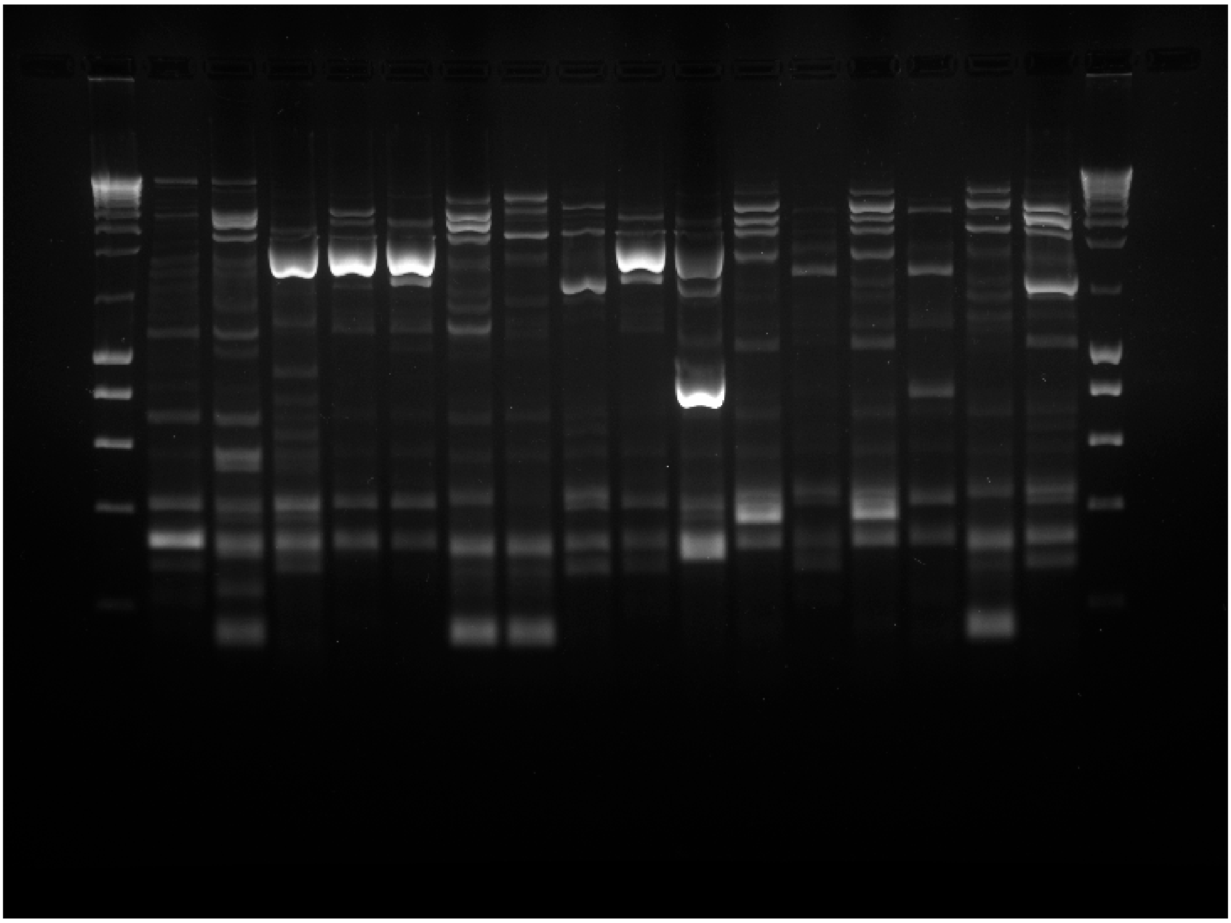

Figure S1. Full scan of the entire original gel in Figure 2 representing ERIC-PCR fingerprinting profiles of 16 *E. coli* isolates under study.

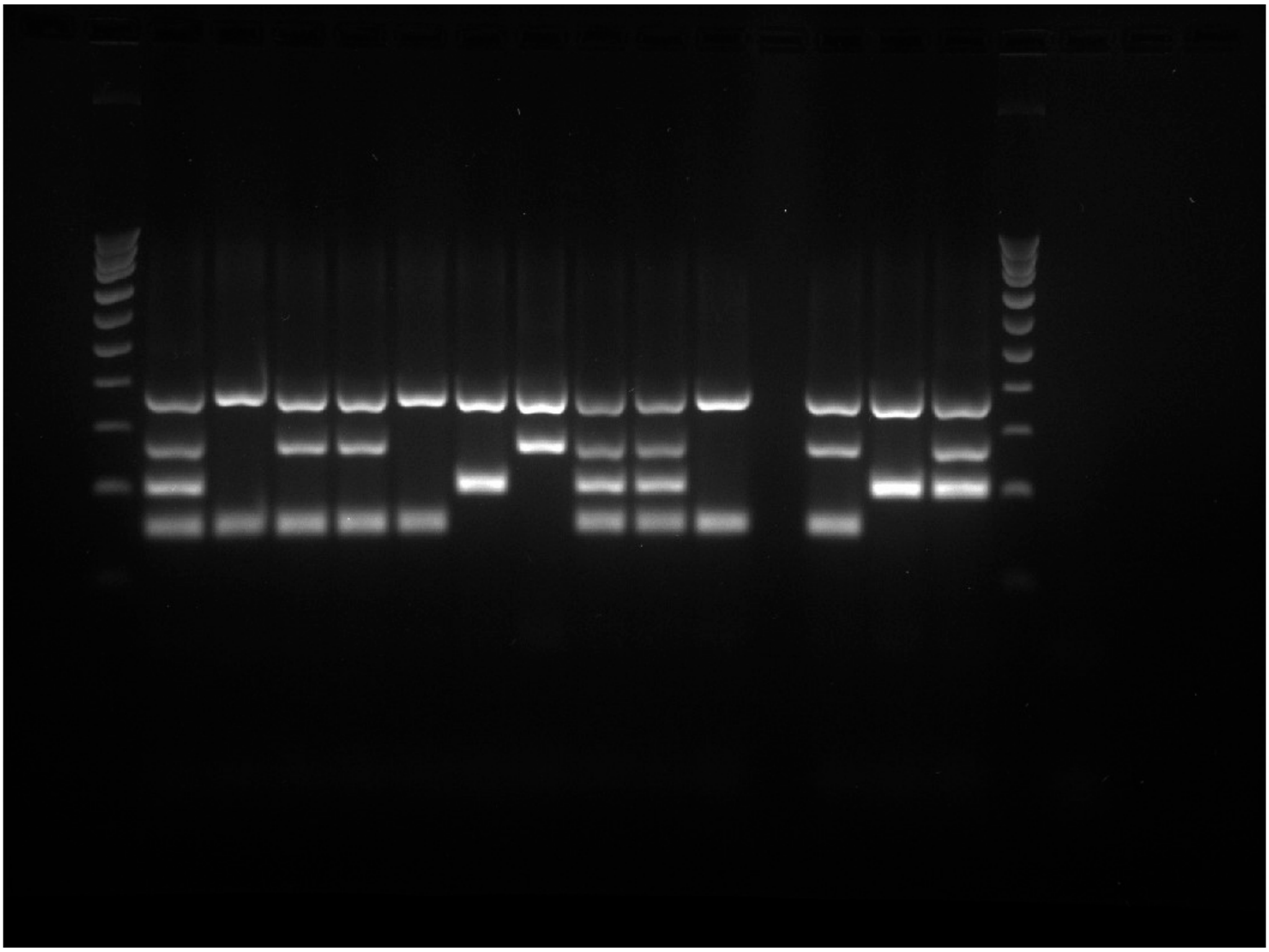

Figure S2. Full scan of the entire original gel in Figure 4 representing quadruplex PCR profiles of 10 *E. coli* isolates.

Table S1. Data on the sampled animals (n=27).

Sample ID	Sex	Species	Age	Collection Date	Sampling District
1	F	*Parabuteo unicinctus*	Adult	29/12/2022	Lisboa
2	F	*Parabuteo unicinctus*	Adult	29/12/2022	Lisboa
3	M	*Parabuteo unicinctus*	Adult	29/12/2022	Lisboa
4	M	*Parabuteo unicinctus*	Juvenile	29/12/2022	Lisboa
5	M	*Parabuteo unicinctus*	Adult	29/12/2022	Lisboa
6	M	*Parabuteo unicinctus*	Adult	08/01/2023	Santarém
7	M	*Falco tinnunculus*	Adult	08/01/2023	Santarém
8	F	*Falco tinnunculus*	Adult	08/01/2023	Santarém
9	?	*Bubo virginianus*	Adult	08/01/2023	Faro
10	?	*Bubo bengalensis*	Adult	08/01/2023	Faro
11	M	*Bubo bubo*	Adult	08/01/2023	Faro
12	?	*Tyto alba*	Adult	08/01/2023	Faro
13	M	*Bubo bubo*	Adult	08/01/2023	Faro
14	M	*Parabuteo unicinctus*	Adult	08/01/2023	Faro
15	F	*Parabuteo unicinctus*	Adult	08/01/2023	Faro
16	M	*Parabuteo unicinctus*	Juvenile	08/01/2023	Faro
17	?	*Ptilopsis leucotis*	Adult	08/01/2023	Faro
18	M	*Urubu aura*	Adult	08/01/2023	Faro
19	?	*Falco tinnunculus*	Juvenile	08/01/2023	Faro
20	F	*Parabuteo unicinctus*	Adult	29/12/2022	Lisboa
21	M	*Parabuteo unicinctus*	Juvenile	29/12/2022	Setúbal
22	F	*Bubo bubo*	Juvenile	29/12/2022	Setúbal
23	M	*Bubo bubo*	Adult	29/12/2022	Setúbal
24	F	*Falco biarmicus*	Juvenile	29/12/2022	Setúbal
25	F	*Falco tinnunculus*	Juvenile	29/12/2022	Setúbal
26	F	*Tyto alba*	Juvenile	29/12/2022	Setúbal
27	F	*Strix aluco*	Adult	29/12/2022	Setúbal

Female (F), Male (M), No data (?).

Table S2. Results of the Isolates' Virulence (n=84) and Resistance Profiles and Phylogenetic Group (n=33).

Isolate	Virulence Profile		Resistance Profile				
	Positive Results	Virulence index	I	R	MAR index	ESBL	Phylogenetic Group
1.3	G; BW	0.333	CL	-	0	Negative	B2
2.1	G; BM	0.333	-	TE; DO	0.222	Negative	B1
2.3	G	0.167	CL; ENR	PRL	0.111	Negative	D
3.1	G	0.167	CL; ENR	-	0	Negative	D
3.4	G	0.167	AMC; CL	TE; ENR; M; DO; SXT; PRL	0.667	Negative	B1
5.2	G	0.167	-	PRL	0.111	Negative	A
6.1	G; BW	0.333	-	-	0	Negative	D
6.2	G; BS	0.333	AMC	TE; CL; DO; PRL	0.444	Negative	B2
6.4	G	0.167	ENR	TE; CL; DO; PRL	0.444	Negative	B2
8.1	G; BM	0.333	AMC; CL	-	0	Negative	B1
11.3	G; BM	0.333	CL	-	0	Negative	B1
12.2	G; BW	0.333	-	-	0	Negative	D
12.3	G; BS	0.333	CL	PRL	0.111	Negative	B2
13.1	-	0	AMC; CL	ENR; M; PRL	0.333	Negative	D
13.4	G; BW	0.333	-	TE; DO; PRL	0.333	Negative	A
14.1	G; BS	0.333	CL	-	0	Negative	B2
14.2	G; BS	0.333	CL	-	0	Negative	B2
14.3	G; BS	0.333	-	-	0	Negative	B2
15.2	G; BM	0.333	CL	-	0	Negative	B1
15.3	G	0.167	CL	TE; ENR; M; DO; SXT; PRL	0.667	Negative	D
16.1	G	0.333	-	CL; PRL	0.222	Positive	D
16.4	G	0.333	-	CL; PRL	0.222	Positive	D
17.2	G	0.167	ENR	TE; DO; SXT; PRL	0.444	Negative	A
17.4	G	0.167	DO	TE; SXT; PRL	0.333	Negative	A
18.2	G; BS	0.333	CL	TE; DO	0.222	Negative	B1
19.3	H; G; BS	0.500	AMC; CL	TE; DO; CN; PRL	0.444	Negative	B1
21.2	G	0.167	CL	-	0	Negative	B1
21.3	G	0.167	-	TE; DO	0.222	Negative	B2
22.1	H; G	0.333	ENR	-	0	Negative	D
26.1	H; G	0.333	CL	-	0	Negative	D
26.2	H	0.167	CL	-	0	Negative	D
26.3	H	0.167	CL	-	0	Negative	D
27.3	H	0.167	CL	-	0	Negative	B2

Haemolysin production (H); Gelatinase activity (G); Biofilm weak production (BW); Biofilm moderate production (BM); Biofilm strong production (BS); Resistance (R); Intermediate susceptibility (I); Tetracycline (TE); Gentamicin (CN); Amoxicillin/Clavulanic Acid (AMC), Cephalexin (CL), Enrofloxacin (ENR), Trimethoprim/Sulfamethoxazole (SXT); Piperacillin (PRL); Doxycycline (DO); Marbofloxacin (M); Extended Spectrum β-Lactamase production (ESBL); Not found (-).
